# Gene expression profiling of colorectal adenomas and early invasive carcinomas by cDNA array analysis

**DOI:** 10.1038/sj.bjc.6602442

**Published:** 2005-03-22

**Authors:** K Nosho, H Yamamoto, Y Adachi, T Endo, Y Hinoda, K Imai

**Affiliations:** 1 First Department of Internal Medicine, Sapporo Medical University, S-1, W-16, Chuo-ku, Sapporo 060-8543, Japan; 2 Department of Clinical Laboratory Science, Yamaguchi University School of Medicine, Ube 755-8505, Japan

**Keywords:** colorectal adenoma, early invasive colorectal carcinoma, cDNA array

## Abstract

It is generally accepted that most colorectal carcinomas arise in pre-existing adenomas. Morphologically, colorectal adenomas can be divided into two groups, protruded type and flat type. The aim of this study was to clarify relevant alterations of gene expression associated with the early stage of colorectal carcinogenesis. Using cDNA array, we analysed the expression profiles of 550 cancer-related genes in 36 colorectal adenomas (18 flat-type and 18 protruded-type adenomas) and 14 early invasive carcinomas. Among the 550 genes, we chose 32 genes the average expression levels of which were at least three-fold up- or downregulated in tumour tissues compared with levels in matched normal tissues. A total of 13 and 19 genes were identified as up- and downregulated genes in tumour tissues, respectively. Among the upregulated genes, the average expression levels of E1AF, bone morphogenic protein (BMP)-4, insulin-like growth factor (IGF)-2, inducible nitric oxide synthase (iNOS), tissue inhibitors of metalloproteinase (TIMP)-1, Smad4, and nm23 in tumour tissues were over five times higher than those in matched normal tissues. Colorectal adenomas and early invasive carcinomas were divided into two major clusters by clustering analysis. Moreover, flat- and protruded-type adenomas were divided into two major clusters by clustering analysis. The expression profiles obtained by the cDNA array clearly indicate that colorectal adenomas and early invasive carcinomas have specific expression profiles. Likewise, the gene expression profiles of flat- and protruded-type adenomas are different. These results indicate that molecular classification of early colorectal tumours by a cDNA array is feasible.

Colorectal carcinoma is one of the most common human malignancies in the world. Although alternative pathways exist, it is generally accepted that most colorectal carcinomas arise in pre-existing adenomas (Jass *et al*, 2002). Despite a large number of studies, little is known about molecular alterations associated with the heterogeneity of colorectal carcinomas.

Morphologically, early colorectal tumors can be divided into two groups, protruded type and flat type. Recently, flat-type colorectal tumours have been reported not only in Japan (Sakashita *et al*, 2000) but also in Western countries (Olschwang *et al*, 1998). Previous studies showed that flat-type colorectal tumours tended to reach deeper layers earlier and to show higher rates of lymphatic invasion and lymph node metastasis than did protruded-type tumours (Kuramoto and Oohara, 1989; Mueller *et al*, 1998). Moreover, it has been thought that some flat-type cancers correspond to *de novo* cancers, which contain no observable adenomatous component and may develop through a distinct genetic pathway (Yashiro *et al*, 2001). Thus, it would be interesting to examine gene expression profiles of colorectal adenomas and early invasive colorectal carcinomas, because comparison of these two groups of tumours will provide information about genes that play an important role during progression from adenoma to carcinoma.

DNA array technology enables measure of the mRNA expression levels of thousands of genes in a single assay. Mainly advanced cancer has been analysed in gene expression profiling-based studies on colorectal cancer (Alon *et al*, 1999; Backert *et al*, 1999; Hegde *et al*, 2001; Kitahara *et al*, 2001; Notterman *et al*, 2001; Takemasa *et al*, 2001; Agrawal *et al*, 2002; Birkenkamp-Demtroder *et al*, 2002; Lin *et al*, 2002; Zou *et al*, 2002; Frederiksen *et al*, 2003; Muro *et al*, 2003; Tureci *et al*, 2003; Williams *et al*, 2003; Bertucci *et al*, 2004). Only a small number of adenoma and early invasive cancer tissues have been analysed in previous studies, and the issue of flat- and protruded-type adenoma tissues has not been directly addressed (Notterman *et al*, 2001; Agrawal *et al*, 2002; Lin *et al*, 2002; Williams *et al*, 2003).

In this study, we therefore applied cDNA array technology to analyse the gene expression profiles of 36 colorectal adenomas (18 flat-type and 18 protruded-type adenomas) and 14 early invasive carcinomas. This is the first study showing molecular classification of early colorectal tumours by a cDNA array analysis.

## MATERIALS AND METHODS

### Patients and tissue samples

A total of 50 paired specimens of colorectal tumour and nontumour tissues were obtained by polypectomy or surgical treatment. These tumour samples consisted of 36 colorectal adenomas and 14 early invasive carcinomas (pT1 in the TNM classification of the Union International Contre Cancer). Normal epithelial tissue samples and tumour tissue samples were carefully macrodissected by expert pathologists. In case of early invasive carcinomas, tumour tissue samples were taken from the macroscopically visible deepest invading part of the tumour after surgical resection. Each tissue specimen was divided into two pieces after resection. For total RNA extraction, one sample was immediately frozen in liquid nitrogen at the time of surgery and stored at −80°C until extraction. The other sample was processed for pathological examination using haematoxylin and eosin staining for the evaluation of the tumour cell content. Only specimens containing more than 80% tumour cells were used for analysis (Horiuchi *et al*, 2003). The histopathological features of the carcinoma specimens were classified according to the TNM classification system. Locations of the colorectal tumours were divided into proximal colon (caecum and ascending and transverse colon) and distal colon (descending and sigmoid colon and rectum). Macroscopic types were divided into protruded type (height of tumour ⩾3 mm) and flat type (height of tumour <3 mm). It was difficult to divide early invasive carcinoma into protruded type or flat type because colorectal tumours become thick when they have invaded the submucosal layer. Therefore, macroscopic type was classified in only colorectal adenomas. The clinicopathological characteristics of colorectal tumours are shown in Table 1. Informed consent was obtained from each subject, and the institutional review committee approved this study.

### cDNA array analysis

Total RNA was extracted from specimens using the acid guanidinum thiocyanate–phenol–chloroform extraction method and treated with DNase I. Biotin-labelled cDNA targets were made from 2.5 *μ*g of total RNA using Gene Navigator cDNA amplification system (Toyobo, Osaka, Japan), including random 9-mer, biotin-16-dUTP, and ReverTraAceTM reverse transcriptase. Free biotin-16-dUTP in the reaction was removed by ethanol precipitation. Gene Navigator cDNA array filter (human cancer, Toyobo) consisted of 550 cancer-related genes and 11 housekeeping genes in duplicate. A complete list is available on the Internet (http://www.toyobo.co.jp). Hybridisation was performed overnight at 68°C in PerfectHybTM (Toyobo). Filters were washed three times in 2 × SSC/0.1% SDS at 68°C for 10 min each, followed by three washes in 0.1 × SSC/0.1% SDS at 68°C for 10 min each. Specific signals on the filters were detected by the chemiluminescence detection kit (Imaging highTM, Toyobo). CDP-Star was used as the chemiluminescent substrate. Quantitative assessment of the signals on the filters was performed by scanning on a Fluor-S MultiImager System (Bio-Rad, Richmond, CA, USA) followed by image analysis using ImaGene software (BioDiscovery, Los Angeles, CA, USA). The data were analysed with normalisation to glyceraldehyde-3-phosphate dehydrogenase (GAPDH) expression. The average of three experiments was calculated.

### Statistical analysis

Expression of each target gene was assessed for associations with clinicopathological characteristics using Mann–Whitney *U*-test for average tumour expressions.

## RESULTS

### cDNA array analysis

To clarify relevant alterations of gene expression associated with early colorectal carcinogenesis, we analysed the gene expression profiles of tumour tissues by a cDNA array (Yamamoto *et al*, 2002). Gene Navigator cDNA array filter (human cancer, Toyobo) consisted of 550 cancer-related genes and 11 housekeeping genes in duplicate. Among the 550 genes the expression profiles of which were analysed, we chose 32 genes the average expression levels of which were at least three-fold up- or downregulated in 50 tumour tissues compared with levels in 50 matched normal tissues. Besides, since many colorectal tumours have been demonstrated to have a diversity of gene expression profiles, we examined the ratios of the selected 32 genes in all 50 tumour/normal pairs individually. The selected genes were classified as commonly changed if their ratio was three-fold up- or downregulated in more than one-third (15 of 50) of the patients. Among the selected 32 genes, all genes satisfied these criteria. A total of 13 genes (insulin-like growth factor (IGF)-2 bone morphogenic protein (BMP)-4, ECGF-1, E1AF, FAK, Rho GDI*β*, nm23, tissue inhibitors of metalloproteinase (TIMP)-1, GSTP1, GST-II, Smad4, inducible nitric oxide synthase (iNOS), and c-jun) and 19 genes (Egr-2, PMS1, Eph, gp130, Rho 8, Ras-GAP, p120, GAK, Erk1, Lamin *β*3, Cdc42, *α*N-catenin, MMP-15, Galectin-1, HLA-DQ, MUC-2, MDR1, Mucin3, and p21) were identified as up- and downregulated genes in tumour tissues, respectively. These genes were associated with transcription (Egr-2 and E1AF), DNA repair or protection (PMS1, GSTP1, and GST-II), cell signalling (Eph, gp130, Rho 8, Ras-GAP, p120, GAK, Rho GDI*β*, Erk1, FAK, and Lamin *β*3), cell cycle (Cdc42), growth factor (IGF-2, BMP-4, and ECGF-1), tumour suppressor (nm23 and Smad4), oncogene (c-jun), cell adhesion (*α*N-catenin), extracellular matrix-degrading enzymes (MMP-15, TIMP-1, and Galectin-1), human leucocyte antigen (HLA-DQ), glycoprotein (MUC-2, MDR1, and Mucin3), CDK inhibitor (p21), and angiogenesis (iNOS).

**Figure 1:**
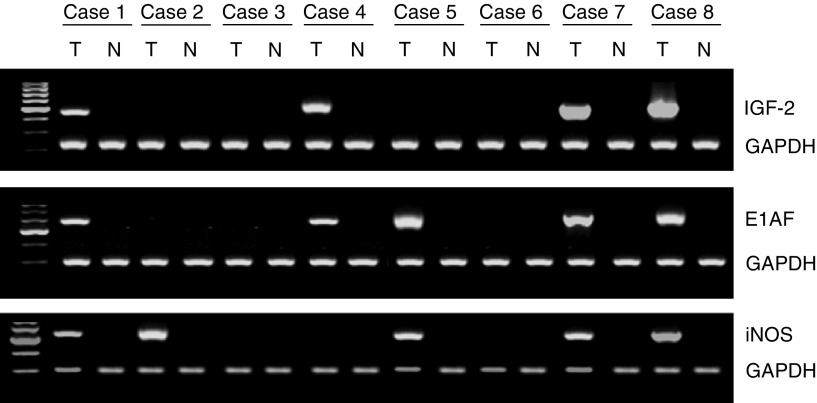
Reverse transcriptase–polymerase chain reaction analysis of mRNA expression for IGF-2, E1AF, and iNOS in colorectal tumour tissues. T and N, matched samples from tumour and nontumour tissue, respectively. Cases 1–4 are colorectal adenomas and cases 5–8 are colorectal carcinomas (pT1).

Among the 13 genes the average expression levels of which were at least three-fold upregulated in tumour tissues compared with levels in matched normal tissues, the average expression levels of E1AF, BMP-4, IGF-2, iNOS, TIMP-1, Smad4, and nm23 genes in tumour tissues were over five times higher than those in matched normal tissues (Table 2). Semiquantitative reverse transcriptase–polymerase chain reaction (RT–PCR) analysis of these differentially expressed genes gave results consistent with those by a cDNA array analysis (Figure 1).

**Figure 2:**
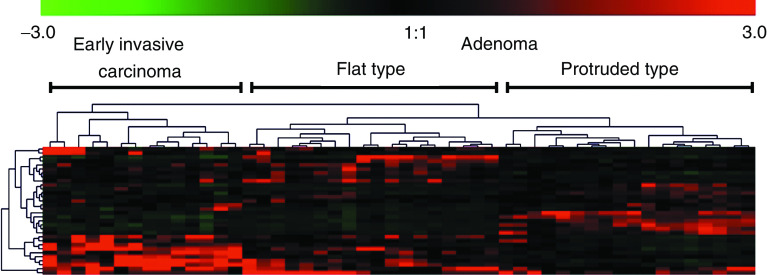
A two-dimensional hierarchical clustering of 32 genes across 50 colorectal tumours. The colour in each well represents relative expression of each gene (vertical axis) in each paired sample (horizontal axis); red, increased in tumour tissues; green, decreased in the tumour tissues. In the sample axis, early invasive carcinomas and adenomas were separated into two different trunks. In the gene axis, 32 genes were clustered in different branches according to their similarity; the shorter the branches, the greater the similarity. In adenomas, subclusters of flat type and protruded type were selected for further analysis (see Figure 3).

Colorectal adenomas (*n*=36) and early invasive carcinomas (*n*=14) were divided into two major clusters by clustering analysis (Figure 2). The average expression levels of 10 (IGF-2, E1AF, iNOS, Rho GDI*β*, GSTP1, c-jun, ECGF1, nm23, Smad4, and TIMP-1) of the 32 genes were significantly higher in the early invasive carcinoma group than in the adenoma group. On the other hand, the average expression levels of 12 (Eph, gp130, GST-II, Rho 8, MUC-2, Ras-GAP, p120, MDR1, *α*N-catenin, Egr-2, PMS1, and GAK) of the 32 genes were significantly lower in the early invasive carcinoma group than in the adenoma group (Table 3).

**Figure 3:**
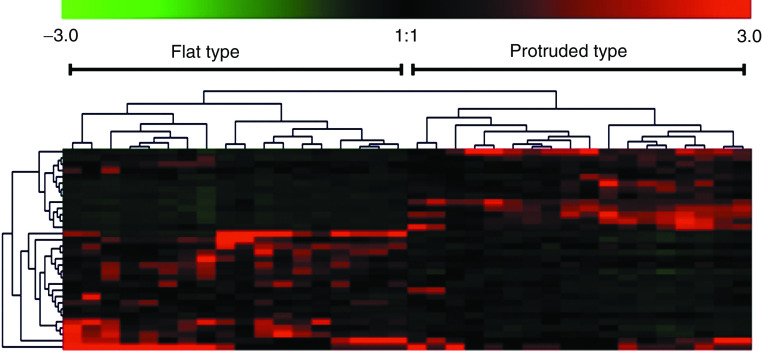
A two-dimensional hierarchical clustering of 32 genes across 36 colorectal adenomas. The colour in each well represents relative expression of each gene (vertical axis) in each paired sample (horizontal axis); red, increased in adenoma tissues; green, decreased in adenoma tissues. In the sample axis, flat- and protruded-type adenomas were separated into two different trunks.

Flat-type (*n*=18) and protruded-type (*n*=18) adenomas were divided into two major clusters by clustering analysis (Figure 3). The average expression levels of 16 (PMS1, nm23, p21, FAK, Smad4, c-jun, ECGF-1, Erk1, GAK, GSTP1, IGF-2, Laminin *β*-3, MMP-15, Mucin3, Rho GDI*β*, and TIMP-1) of the 32 genes were significantly higher in the flat-type group than in the protruded-type group, while the average expression levels of 11 (HLA-DQ, Cdc42, Egr-2, Eph, Galectin-1, gp130, GST-II, MDR1, p120, Ras-GAP, and Rho 8) of the 32 genes were significantly lower in the flat-type group than in the protruded-type group (Table 4).

Among the 32 genes, the average expression levels of eight genes (Rho GDI*β*, c-jun, iNOS, TIMP-1, GSTP1, ECGF1, nm23, and Smad4) and four genes (MUC-2, *α*N-catenin, PMS1, and GAK) were significantly lower and higher, respectively, in both the flat- and protruded-type adenoma groups than in the early invasive carcinoma group. The average expression levels of two genes (Cdc42 and Galectin-1) and four genes (p21, Erk1, Mucin 3, and Laminin *β*-3) were significantly lower and higher, respectively, in the flat-type adenoma group than those in the early invasive carcinoma group (Table 5). On the other hand, the average expression levels of seven genes (Erk1, Mucin 3, Laminin *β*-3, IGF-2, FAK, MMP-15, and E1AF) and nine genes (Cdc42, Eph, gp130, GST-II, Rho 8, Ras-GAP, p120, MDR1, and Egr-2) were significantly lower and higher, respectively, in the protruded-type adenoma group than in the early invasive carcinoma group (Table 5).

## DISCUSSION

Using a cDNA array, we analysed the gene expression profiles of 36 colorectal adenoma and 14 early invasive carcinoma tissues to clarify characteristic changes associated with the early stage of colorectal carcinogenesis. The reason why we chose early invasive carcinoma is that it represents the early stage of colorectal carcinoma. Among the 550 genes the expression profiles of which were analysed, we chose 32 genes the average expression levels of which were at least three-fold up- or downregulated in 50 tumour tissues compared with levels in 50 matched normal tissues. Among the 13 upregulated genes, the expression levels of E1AF, BMP-4, IGF-2, iNOS, TIMP-1, Smad4, and nm23 genes were over five-times higher than those in matched normal tissues.

E1AF (human PEA3/ETV4) is an ets family transcriptional factor. We recently reported that E1AF plays a key role in the progression of colorectal carcinoma (Horiuchi *et al*, 2003). Thus, our results of cDNA array analysis extend roles of E1AF in the late stage to early stage of colorectal carcinogenesis. BMP-4 is a member of the TGF-*β* superfamily of growth factors. It has been reported that BMP-4 is overexpressed and secreted by human colon cancer cells with mutant APC genes (Kim *et al*, 2002). Our results suggest that BMP-4 overexpression plays an important role in the early stage of colorectal carcinogenesis. iNOS has been reported to play a crucial role in cancer development by promoting angiogenesis (Jenkins *et al*, 1995). Our results are consistent with those of previous studies showing an important role of iNOS in the early stage of colorectal carcinogenesis (Xu *et al*, 2003). Interestingly, nitric oxide (NO), generated by iNOS, reportedly augments the synergistic interaction between E1AF and its transcription coactivator CBP/p300, resulting in the facilitation of induction of tumour-related genes, such as COX-2 (Liu *et al*, 2004).

Several lines of evidence suggest that IGF-2 plays an important role in the progression of colorectal tumours (Lambert *et al*, 1990). Moreover, expression of IGF-2 protein has been reported to be associated with advanced tumour stage and poor survival (Kawamoto *et al*, 1998; Peters *et al*, 2003). It has also been suggested that IGF-2 plays a role in the development of liver metastasis from colorectal cancer (Kawamoto *et al*, 1999). Thus, our results extend roles of IGF-2 in the late stage to early stage of colorectal carcinogenesis.

Among the four known tissue inhibitors of metalloproteinases (TIMPs), TIMP-1 has functions apart from its protease inhibitory action. Several investigators have reported that TIMP-1 has growth-promoting properties and might also stimulate tumour growth by inhibiting apoptosis (Holten-Andersen *et al*, 2002). Overexpression of TIMP-1 has been reported in colorectal cancer tissues (Murashige *et al*, 1996). Holten-Andersen *et al* (2005) recently reported that TIMP-1 mRNA was detected in all of 24 cases of colorectal cancer tissues by *in situ* hybridisation, but it was detected in only two of seven adenoma tissues. In the current study, we also found that the average expression levels of TIMP-1 were significantly higher in the early invasive carcinoma group than in the adenoma group. Besides, levels of TIMP-1 in blood were significantly elevated in colorectal cancer patients compared to healthy donors, and high plasma TIMP-1 levels were associated with short survival of colorectal cancer patients (Holten-Andersen *et al*, 2005). Therefore, TIMP-1 appears to be a novel marker for detection of early colorectal cancer and for prognostic stratification of colorectal cancer patients.

Smad4 is an intracellular transmitter of TGF-*β* signals and its tumour suppressor function is presumed to reside in its capacity to mediate TGF-*β*-induced growth inhibition. However, there is accumulating evidence that this hypothesis may be too simple (Muller *et al*, 2002). Although functional inactivation of Smad4 in colorectal cancer frequently occurs at late stages when tumours acquire invasive and metastatic capabilities, the roles of TGF-*β* signals in carcinogenesis are complex and also comprise tumour-promoting functions in colorectal carcinogenesis (Muller *et al*, 2002; Reinacher-Schick *et al*, 2004). Nevertheless, the reason for the overexpression of Smad4 in early colorectal tumour tissues remains unknown. It may be induced to inhibit tumour growth by some compensatory mechanisms. Further analysis is needed to clarify this issue.

The nm23 gene was first identified as a gene the expression level of which was reduced in highly metastatic rodent tumours relative to poorly metastatic tumour cells (Steeg *et al*, 1988). The transfection of nm23 cDNA into various cancer cell lines resulted in suppression of the metastatic potential of motility, invasion, or colonisation (Suzuki *et al*, 2004). However, Bertucci *et al* (2004) recently reported over- and downexpression of nm23 in colorectal cancer tissues and in those with poor prognosis, respectively. The reason why nm23 gene was highly expressed in tumour tissues in the current study may be due to the fact that all tumour samples consisted of early colorectal tumours without metastasis. Further analysis is needed to clarify this issue.

We identified 22 genes the expression levels of which differed significantly in colorectal adenomas and early invasive carcinomas. Colorectal adenomas and early invasive carcinomas were divided into two major clusters by clustering analysis. This result is consistent with that of a recent study showing that nine colorectal adenomas were separated from 11 differentiated colorectal carcinomas by using oligonucleotide arrays (Lin *et al*, 2002). The expression profiles obtained by our cDNA array demonstrated that colorectal adenomas and early invasive carcinomas have specific expression profiles. Among the seven upregulated genes the expression levels of which were over five times higher than those in the matched normal tissues, the expression levels of IGF-2, E1AF, iNOS, nm23, Smad4, and TIMP-1 genes were significantly higher in the early invasive carcinoma group than in the adenoma group. These results suggest that these genes play an important role in the progression from adenoma to carcinoma.

On the other hand, GAK was the most downregulated gene in the early invasive carcinoma group relative to the adenoma group. GAK is a serine/threonine kinase that shows high homology outside its kinase domain with auxilin. Like auxilin, GAK has been shown to be a cofactor for uncoating clathrin vesicles *in vitro*. Zhang *et al* (2004) reported that downregulation of GAK by small hairpin RNA increased the levels of epidermal growth factor (EGF) receptor expression and tyrosine kinase activity, resulting in a large increase in the levels of activated extracellular signal-regulated kinase 5 and Akt. Moreover, downregulation of GAK has been reported to result in outgrowth of monkey kidney CV1P cells in soft agar, raising the possibility that loss of GAK function may promote tumorigenesis. Thus, our results suggest that downregulation of GAK plays an important role in the progression from colorectal adenoma to carcinoma.

The adenoma–carcinoma sequence (ACS) is widely accepted as a pathogenesis of colorectal carcinoma. A multistep genetic model for colorectal carcinogenesis based on the ACS has been proposed (Vogelstein *et al*, 1988). In the ACS sequence, mutations in the K-ras gene and various tumour suppressor genes, such as APC and *p*53, are known to accumulate during the progression from normal to malignant tissue. Although coexistence of all three mutations has been reported to be a rare occurrence (Smith *et al*, 2002), the majority of sporadic colorectal carcinomas are still thought to develop and progress through this pathway. It has been thought that *de novo* cancers develop from normal colonic mucosa directly. However, critical genetic abnormality is not known. Most protruded-type colorectal cancers have adenomatous elements in the periphery when found at an early stage, suggesting that these cancers have arisen from pre-existing adenomas. On the other hand, adenomatous components are not detectable microscopically in some flat-type cancers, suggesting that flat-type cancers correspond to *de novo* cancer (Yashiro *et al*, 2001). The reason why we could not detect any changes in the expression of APC, *p*53, and K-ras genes may be due to the fact that mutations of these genes do not necessarily result in alterations of mRNA expression levels.

In the current study, flat- and protruded-type adenomas were divided into two major clusters by clustering analysis. We identified 27 genes the expression levels of which differed significantly in flat- and protruded-type adenomas. The expression levels of 16 genes (PMS1, nm23, p21, FAK, Smad4, c-jun, ECGF-1, Erk1, GAK, GSTP1, IGF-2, Laminin *β*-3, MMP-15, Mucin3, Rho GDI*β*, and TIMP-1) were significantly higher in the flat-type group than in the protruded-type group. On the other hand, the expression levels of 11 genes (HLA-DQ, Cdc42, Egr-2, Eph, Galectin-1, gp130, GST-II, MDR1, p120, Ras-GAP, and Rho 8) were significantly lower in the flat-type group than in the protruded-type group.

Among the 18 genes the expression levels of which were significantly different in the early invasive carcinoma group and the flat-type adenoma group, the expression levels of eight genes (p21, MUC-2, Erk1, Mucin 3, *α*N-catenin, PMS1, Lamin *β*3, and GAK) and 10 genes (Rho GDI*β*, c-jun, Cdc42, iNOS, Galectin-1, TIMP-1, GSTP1, ECGF1, nm23, and Smad4) were significantly higher and lower, respectively, in the flat-type adenoma group than in the early invasive cancer group. On the other hand, among the 28 genes the expression levels of which were significantly different in the early invasive carcinoma group and the protruded-type adenoma group, the expression levels of 13 genes (GAK, MUC-2, PMS1, *α*N-catenin, Cdc42, Eph, gp130, GST-II, Rho 8, Ras-GAP, p120, MDR1, and Egr-2) and 15 genes (iNOS, FAK, MMP-15, E1AF, IGF-2, Laminin *β*3, Smad4, TIMP1, nm23, Rho GDI*β*, GSTP1, c-jun, ECGF1, Erk1, and Mucin 3) were significantly higher and lower, respectively, in the protruded-type adenoma group than in the early invasive cancer group. These results suggest that flat- and protruded-type adenomas have specific expression profiles and that genes that play a crucial role in the progression from each type of adenoma to carcinoma are different.

In conclusion, the expression profiles obtained by the cDNA array clearly indicate that colorectal adenomas and early invasive carcinomas have specific expression profiles. Likewise, the gene expression profiles of flat- and protruded-type adenomas are different. These results indicate that molecular classification of early colorectal tumours by a cDNA array is feasible.

## Figures and Tables

**Table 1:** Clinicopathological characteristics of patients with colorectal tumour

		**Adenoma (***n***=36)**	
**Characteristics**	**Early invasive carcinoma (*n*=14)**	**Protruded type (*n*=18)**	**Flat type (*n*=18)**
Age (years, mean±s.d.)	69.1±7.3	67.9±5.8	71.1±4.9
Size (mm, mean±s.d.)	25.7±10.0	10.5±5.4	14.2±12.7
Gender			
Male	8	11	13
Female	6	7	5
Location			
Proximal	9	5	10
Distal	5	13	8

**Table 2:** Average expression levels of E1AF, BMP4, IGF-2, iNOS, TIMP-1, Smad4, and nm23 genes in tumour tissues were over five times higher than those in the matched normal tissues

**Gene name**	**Function**	**T average/N average**
E1AF	Transcription	8.11
BMP4	Growth factor	7.64
IGF-2	Growth factor	6.80
iNOS	Angiogenesis	6.55
TIMP-1	Inhibitor of MMPs	6.39
Smad4	Tumour suppressor	6.03
nm23	Tumour suppressor	5.33

T average=average expression levels in tumour tissues; N average,=average expression levels in matched normal tissues. The average of three experiments is shown.

**Table 3:** Genes the expression levels of which differed significantly in the early invasive carcinoma group and the adenoma group (Mann–Whitney *U*-test)

**Gene name**	**Accession no.**	**Gene function**	***P*-value**	
Eph	M18391	Cell signalling	0.0342	CA<AD
Gp130	M57230	Cell signalling	0.0122	CA<AD
GST-II	U77604	DNA repair or protection	0.0108	CA<AD
Rho 8	X95282	Cell signalling	0.0069	CA<AD
MUC-2	M74027	Glycoprotein	0.0046	CA<AD
Ras-GAP	AF051311	Cell signalling	0.0035	CA<AD
P120	AF062324	Cell signalling	0.0029	CA<AD
MDR1	AF016535	Glycoprotein	0.0019	CA<AD
*α*N-catenin	M94151	Cell adhesion	0.0014	CA<AD
Egr-2	X53700	Transcription	0.0011	CA<AD
PMS1	U13695	DNA repair or protection	0.0002	CA<AD
GAK	D88435	Cell signalling	0.0001	CA<AD
IGF-2	M29645	Growth factor	0.0491	CA>AD
E1AF	D12765	Transcription	0.0187	CA>AD
iNOS	AB022318	Angiogenesis	0.0084	CA>AD
Rho GDI*β*	L20688	Cell signalling	0.0002	CA>AD
GSTP1	X06547	DNA repair or protection	<0.0001	CA>AD
c-jun	J04111	Oncogene	<0.0001	CA>AD
ECGF1	M63193	Growth factor	<0.0001	CA>AD
nm23	X17620	Tumour suppressor	<0.0001	CA>AD
Smad4	U44378	Tumour suppressor	<0.0001	CA>AD
TIMP1	X03124	Extracellular matrix-Degrading enzymes	<0.0001	CA>AD
BMP-4	D30751	Growth factor	0.7494	
Cdc42	M57298	Cell cycle	0.65	
Erk1	X60188	Cell signalling	0.2101	
FAK	L13616	Cell signalling	0.0878	
Galectin-1	J04456	Extracellular matrix-degrading enzymes	0.136	
HLA-DQ	U77589	Human leucocyte antigen	0.8289	
Laminin *β*-3	D37766	Cell signalling	0.7134	
MMP-15	Z48482	Extracellular matrix-degrading enzymes	0.8289	
Mucin 3	AF143371	Glycoprotein	0.2897	
P21	U03106	CDK inhibitor	0.1734	

CA=early invasive carcinoma group; AD=adenoma group.

**Table 4:** Genes the expression levels of which differed significantly in the flat-type adenoma group and the protruded-type adenoma group (Mann–Whitney *U*-test)

**Gene name**	**Accession no.**	**Gene function**	***P*-value**	
PMS1	U13695	DNA repair or protection	0.0136	P<F
nm23	X17620	Tumour suppressor	0.0004	P<F
p21	U03106	CDK inhibitor	0.001	P<F
FAK	L13616	Cell signalling	0.0017	P<F
Smad4	U44378	Tumour suppressor	0.0429	P<F
c-jun	J04111	Oncogene	<0.0001	P<F
ECGF1	M63193	Growth factor	<0.0001	P<F
Erk1	X60188	Cell signalling	<0.0001	P<F
GAK	D88435	Cell signalling	<0.0001	P<F
GSTP1	X06547	DNA repair or protection	<0.0001	P<F
IGF-2	M29645	Growth factor	<0.0001	P<F
Laminin *β*-3	D37766	Cell signalling	<0.0001	P<F
MMP-15	Z48482	Extracellular matrix-degrading enzymes	<0.0001	P<F
Mucin 3	AF143371	Glycoprotein	<0.0001	P<F
Rho GDI*β*	L20688	Cell signalling	<0.0001	P<F
TIMP1	X03124	Extracellular matrix-degrading enzymes	<0.0001	P<F
HLA-DQ	U77589	Human leucocyte antigen	0.0003	P>F
Cdc42	M57298	Cell cycle	<0.0001	P>F
Egr-2	X53700	Transcription	<0.0001	P>F
Eph	M18391	Cell signalling	<0.0001	P>F
Galectin-1	J04456	Extracellular matrix-degrading enzymes	<0.0001	P>F
Gp130	M57230	Cell signalling	<0.0001	P>F
GST-II	U77604	DNA repair or protection	<0.0001	P>F
MDR1	AF016535	Glycoprotein	<0.0001	P>F
p120	AF062324	Cell signalling	<0.0001	P>F
Ras-GAP	AF051311	Cell signalling	<0.0001	P>F
Rho 8	X95282	Cell signalling	<0.0001	P>F
E1AF	D12765	Transcription	0.1488	
iNOS	AB022318	Angiogenesis	0.1639	
BMP-4	D30751	Growth factor	0.1966	
MUC-2	M74027	Glycoprotein	0.4107	
*α*N-catenin	M94151	Cell adhesion	0.5478	

F=flat-type adenoma group; P=protruded-type adenoma group.

**Table 5:** Genes the expression levels of which differed significantly in the early invasive carcinoma group and the protruded-type (28 genes) or flat-type (18 genes) adenoma group (Mann-Whitney *U*-test)

**Gene name**	**Accession no.**	***P*-value**		***P*-value**	
BMP-4	D30751	0.6128		0.2968	
Cdc42	M57298	0.001	CA<AD (P)	0.0122	CA>AD (F)
c-jun	J04111	<0.0001	CA>AD (P)	0.0135	CA>AD (F)
E1AF	D12765	0.0086	CA>AD (P)	0.13	
ECGF1	M63193	<0.0001	CA>AD (P)	0.0002	CA>AD (F)
Egr-2	X53700	<0.0001	CA<AD (P)	0.3423	
Eph	M18391	0.0005	CA<AD (P)	0.7903	
Erk1	X60188	<0.0001	CA>AD (P)	0.0276	CA<AD (F)
FAK	L13616	0.0304	CA>AD (P)	0.4033	
GAK	D88435	0.0227	CA<AD (P)	<0.0001	CA<AD (F)
Galectin-1	J04456	0.704		0.0027	CA>AD (F)
Gp130	M57230	<0.0001	CA<AD (P)	0.9093	
GST-II	U77604	<0.0001	CA<AD (P)	0.8197	
GSTP1	X06547	<0.0001	CA>AD (P)	0.0006	CA>AD (F)
HLA-DQ	U77589	0.1489		0.0682	
IGF-2	M29645	0.0034	CA>AD (P)	0.5937	
iNOS	AB022318	0.0402	CA>AD (P)	0.0098	CA>AD (F)
Laminin *β*-3	D37766	0.0016	CA>AD (P)	0.0001	CA<AD (F)
MDR1	AF016535	<0.0001	CA<AD (P)	0.4941	
MMP-15	Z48482	0.025	CA>AD (P)	0.0627	
MUC-2	M74027	0.0044	CA<AD (P)	0.0334	CA<AD (F)
Mucin 3	AF143371	<0.0001	CA>AD (P)	0.0227	CA<AD (F)
nm23	X17620	<0.0001	CA>AD (P)	0.0001	CA>AD (F)
p120	AF062324	<0.0001	CA<AD (P)	0.4941	
p21	U03106	0.7324		0.0402	CA<AD (F)
PMS1	U13695	0.0044	CA<AD (P)	0.0002	CA<AD (F)
Ras-GAP	AF051311	<0.0001	CA<AD (P)	0.7324	
Rho 8	X95282	<0.0001	CA<AD (P)	0.8494	
Rho GDI*β*	L20688	<0.0001	CA>AD (P)	0.0304	CA>AD (F)
Smad4	U44378	<0.0001	CA>AD (P)	<0.0001	CA>AD (F)
TIMP1	X03124	<0.0001	CA>AD (P)	0.0024	CA>AD (F)
*α*N-catenin	M94151	0.0014	CA<AD (P)	0.0151	CA<AD (F)

CA=early invasive carcinoma group; AD (P)=protruded-type adenoma group; AD (F)=flat-type adenoma group.
